# The role of B cells in cancer development

**DOI:** 10.3389/fonc.2022.958756

**Published:** 2022-08-11

**Authors:** Rongying Tan, Manhua Nie, Wang Long

**Affiliations:** ^1^ Clinical Nursing Teaching and Research Section, The Second Xiangya Hospital, Central South University, Changsha, China; ^2^ Department of Kidney Transplantation, The Second Xiangya Hospital of Central South University, Changsha, China; ^3^ Department of Pathology, Nihon University, Tokyo, Japan

**Keywords:** B cell, tumor, tumor immunity, cancer, immunotherapy, tumor microenvironment

## Abstract

B cells play a critical role in adaptive immune responses mainly due to antigen presentation and antibody production. Studies about the tumor-infiltrating immune cells so far demonstrated that the function of B cells in tumor immunity is quite different among various tumor types. The antigen presentation of B cells is mainly anti-tumoral, while the role of antibody production is controversial. Moreover, the immunosuppressive regulatory B cells are detrimental to anti-tumor immunity *via* the secretion of various anti-inflammatory cytokines. This review briefly summarizes the different roles of B cells classified by the primary function of B cells, antigen presentation, antibody production, and immunity regulation. Further, it discusses the potential therapeutic target of B cells in tumor immunity.

## Introduction

Cancer is still a threat to humanity due to its high death rate (1). Various therapies have been developed to treat cancer, including surgery, radiotherapy, chemotherapy, immunotherapy, etc. Among these therapies, immunotherapy has become more and more attractive for researchers, companies, and clinicians in recent years (2). T cell-based immunotherapy is critical and effective in cancer therapy, and the promising outcome of the antibodies targeting immune checkpoints in the treatment of cancer created a grave impact on immunotherapy (2, 3). Though the CD8^+^ T cells have an irreplaceable role in the cytotoxicity in the tumor microenvironment (TME) (4) and immune checkpoint inhibitors (ICIs) are quite efficient in many cancer types, most patients are still resistant to ICIs (5). Increasing studies demonstrated the function of other immune cells in the development of cancer in recent years (6, 7), which might be additional and optimal targets for the treatment of cancer.

B cells are involved in adaptive immunity as the antigen-presenting cells (APCs) and antibody-secreting cells (ASCs), while the function of B cells in cancer immunity is controversial. B cell depletion in mice by anti-IgM treatment from birth showed resistance to syngeneic fibrosarcoma and reduced incidence of pulmonary metastasis (8). In contrast, the lung adenocarcinoma cell inoculation in µMT mice failed to show any difference to WT mice (9), yet the µMT mice had faster tumor growth than WT mice when the tumor cell line was transfected with B cell-specific neoantigen (9). The function of B cells in tumor growth seems to vary among different tumor cell lines. Moreover, antibody production from B cells is not always beneficial. For example, antibody-dependent cellular cytotoxicity (ADCC) is a critical mechanism of the antibody in the anti-tumor effect of B cells (10), while the immune complexes in circulation or TME are correlated with poor clinical outcomes (11).

In this review, we will briefly discuss the immunological mechanism of B cells in cancer immunity to elucidate the controversial phenomenon in various tumor types and potential therapeutic targets of B cells in different tumor types. This review is classified by the basic functions of B cells, but not anti- and pro- tumoral functions of B cells, which is already discussed in other reviews (12).

## Antigen-presenting cells

B cells are efficient APCs in T cell-dependent (TD) antigen-induced humoral immunity. TD antigens are recognized and engulfed by B cells through B cell receptor (BCR), degraded in lysosome, and presented to CD4^+^ cells, resulting in CD4^+^ T cells and further CD8^+^ cells activation (13). Several studies demonstrated the antigen presentation of B cells plays a critical role in tumor-specific CD4^+^ and CD8^+^ T cell activation. B cells undoubtedly present antigen to induce T cell activation in virus-induced tumor growth (14). In the syngeneic B16 melanoma cell line transfer system, B cell depletion by anti-CD20 antibody treatment resulted in a two-fold bigger tumor volume and impaired interferon-γ (IFN- γ) and tumor necrosis factor (TNF-α) production from CD4^+^ T cells and CD8^+^ T cells (15).

A recent study elucidated how antigen presentation of B cells plays a role in tumor immunity. T follicular helper (TFH) cells are involved in B cell maturation and activation. Germinal center (GC) B cells could be activated by TFH-B interaction and further differentiate into short-term living plasma cells, long-term living plasmablasts, and memory B cells. The single-cell RNA sequencing result of tumor-infiltrating lymphocytes in many studies revealed the presence of GC B cells in the TME (9, 16), yet the role of GC B cells is not well known. The study done by Cui et al. in lung adenocarcinoma patients elucidated that GC B cells facilitate the function of CD8^+^ T cells in anti-tumor immunity *via* the TFH-GC B cell interaction in a neoantigen-dependent manner (9). They utilized a lung adenocarcinoma cell line (KP) with limited somatic mutations, which means that there are few or no neoantigen expression and weak B/T cell responses so that B cell or T cell depletion doesn’t affect the tumor growth. With the transfection of HELLO fusion protein, which contains HEL, GP33, and GP66 that can be recognized by MD4 transgenic BCR, GP33-specific CD4 TCR, and GP66-specific CD8 TCR, respectively, KP-HELLO cells are able to activate specific B/T cells. The inoculation of KP-HELLO cells in B cell knockout or TFH knockout mice showed much faster tumor growth and weaker CD8^+^ T cell function compared to tumor growth inoculated in WT mice, suggesting that the GC B cells that recognize the neoantigen and further interact with activated CD4^+^ T cells are able to support CD8^+^ T cells function in TME. Further results demonstrated that interaction between neoantigen-specific TFH and GC B cells and interleukin-21 (IL-21) secreted by TFH cells are necessary for the cytotoxicity of CD8^+^ T cells (9).

## Antibody-secreting cells

B cells play an essential role in the adaptive immune responses by producing antibodies (17). At the same time, the role of antibody-secreting B cells is a double-edged sword in tumor immunity. Once the B cells are activated by recognizing the neoantigen, B cells participate in a two-pathway differentiation process that induces both short-lived plasmablasts and long-lived plasma cells and memory B cells (17). Therefore, these plasmablasts, plasma cells, memory B cells, and the secreted antibodies are neoantigen-specific. Both BCR signaling that provides binding to the antigen, and the B-T cell interaction are essential in the TD antigen-involved long-term antibody production (17).

Commonly, the antibodies are thought to be anti-tumoral. Antibodies with high FcγR affinity and target neoantigens expressed on tumor cell surface induce ADCC, antibody-dependent cellular phagocytosis (ADCP), and complement-dependent cytotoxicity (CDC), which are significant mechanisms of antibody drugs for cancer therapy. For example, the Fc domain of the monoclonal antibody (mAb) has a different affinity to different FcγR expressed on various immune cells (18), among which natural killer (NK) cell is involved in ADCC and is discussed in many mAb treatments in cancer (10, 19). Several mAbs have been used in the clinic based on their cytotoxicities, such as anti-GD2 mAb for melanoma and neuroblastoma treatment (20–23) and chimeric anti-CD20 mAb and anti-CD22 mAb for leukemia treatment (24–27).

Unfortunately, not all of the antibodies contribute to anti-tumoral immunity. Antibodies bind to various antigens released by tumor cells and form circulating immune complexes (CICs), which correlate with poor outcomes (11). In the squamous cell carcinoma mouse model, CICs accumulate in the dermal stroma of neoplastic tissue, activate FcγR on residents, and recruit pro-tumoral and angiogenic myeloid cells (especially mast cells and macrophages) to facilitate tumor cell survival and angiogenesis (28).

Except for IgG, IgA is also a double-edged sword for tumor growth. Many studies have found the accumulation of IgA-producing B cells in TME (29, 30), yet the role of IgA in tumor growth is still controversial. In ovarian cancer patients, tumor-infiltrating B cell-derived IgA dampens tumor growth through the unspecific transcytosis and neoantigen-specific phagocytosis (29). Yet the function of IgA in other cancers is entirely different. Several cancer types have shown that the proportion of IgA-producing cells is highly associated with poor outcomes (31–33). IgA is pro-tumoral in these cases and has the following mechanisms. Firstly, the IgA production is not induced by neoantigen presentation but by the immunosuppressive microenvironment, and the IgA cannot mediate ADCC (34, 35). Secondly, IgA is immunosuppressive in mucosal immunity (36). IgA deficiency leads to a higher risk of inflammation (37–39), and the interaction between IgA and marginal zone B and B1 cell-specific protein (MZB1) may be an important factor (36). What’s more, IgA induces anti-inflammatory cytokine interleukin-10 (IL-10) production from monocytes and further inhibits the immune system (40).

## Regulatory B cells

The discovery of a population of the suppressive function of B cells can be retrospect to 1974 since B cells could delay hypersensitivity (41, 42). Subsequently, more and more papers found that some B cells inhibit the development of various diseases such as experimental autoimmune encephalomyelitis (EAE) (43), allograft rejection (44, 45), lupus nephritis (LN) (46), type 1 diabetes (T1D) (47, 48), anti-neutrophil cytoplasmic antibody (ANCA)-associated vasculitis (AAV) (49) and so on. These B cells regulate immune responses by secreting anti-inflammatory cytokines such as IL-10 (50–54), IL-35 (55–57), and transforming growth factor-β (TGF-β) (58, 59) to dampen CD4^+^ T cells (60), CD8^+^ T cells (53), antibody production (61) and facilitate regulatory T (Treg) cells (62, 63). These B cells are so-called Breg cells. Breg cells are not restricted to a specific B cell phenotype. Therefore, IL-10-producing B cells, for example, are usually utilized to detect Breg cells. Since Breg cells vary in various phenotypes, those types of B cells all have an inhibitory function in immune responses. The phenotype of Breg cells mainly includes transitional B cells (CD19^+^CD24^hi^CD38^hi^) (64) and plasmablasts (CD19^+^CD27^int^CD38^+^) (65) in human, follicular B cells (CD19^+^CD23^+^CD21^int^), marginal zone B cells (CD19^+^CD23^-^CD21^hi^), plasma/plasmablasts (CD19^+^/B220^lo/-^CD138^+^), transitional B cells and B10 cells (CD19^+^/B220^lo/-^CD1d^+^CD5^+^) in mice (50).

Breg cells can not only impair immune responses in TME by secreting antibodies as described above but many anti-inflammatory cytokines production and pathways also contribute to immunosuppression in TME. IL-10 is the most important anti-inflammatory cytokine defining the Breg cells, several pathways are involved in IL-10 production (66, 67). For example, IL-10 production is increased from B cells when stimulated with LPS or CpG (68–70), and MyD88, the downstream of TLR, is necessary for IL-10 production from B cells under LPS stimulation (71), suggesting that TLR activation is able to induce Breg cells differentiation. CD40 and BCR signaling are also related to IL-10 production, as anti-CD40 antibody treatment *in vivo* and *in vitro* expands the IL-10^+^ B cells, and antigen-stimulated B cells transfer in the EAE mouse model rescued IL-10 production in a CD40-dependent manner (72, 73). B cell-derived IL-10 is a strong immunosuppressive cytokine in various autoimmune diseases, it is also important in tumor growth. B cell-deficient mice showed slower tumor growth than WT mice when the mice bearing MC38 carcinoma and EL4 thymoma, and this effect is related to the B cell-derived IL-10 (74, 75). IFN- γ production reduced from B cell-knock out splenic cells when cocultured with WT B cells, and IL-10 production from B cells increased after coculturing with irradiated melanoma cells, not sarcoma cells, indicating that Breg cells suppress the anti-tumor immunity to certain tumors (75). IL-10 production from B cells impairs inflammatory cytokines, including TNF-α and IFN-γ, secretion from cytotoxic T cells to promote tumor growth. While in the chemical carcinogenesis of skin, TNF-α is a promoter for tumor growth, IL-10 produced by B cells facilitates tumor growth in a TNF-α-dependent manner (76). Moreover, IL-10-producing B cells are also being found to promote tumor growth in non-Hodgkin B cell lymphoma (77).

TGF-β is another critical anti-inflammatory cytokine secreted by Breg cells. In the breast tumor model, TGF-β is highly expressed on tumor-infiltrating B cells and associated with the conversion of resting CD4^+^ T cells to Treg cells (78, 79). Furthermore, IL-35 produced by Breg cells also plays a promotion role in pancreatic tumor growth (80, 81). Altogether, Breg cells suppress anti-tumor immunity *via* the secretion of anti-inflammatory cytokines such as IL-10, TGF-β and IL-35.

## Anti- and pro-tumorigenic factors secreted by B cells

Except for the antibodies and cytokines described above, B cells also secrete some other factors that affect tumor growth. Lymphotoxin α1β2 (LTα1β2) plays a critical role in the lymphoid organ development and especially in ectopic tertiary lymphoid organs (82–84). Indeed, the presence of B cells in tertiary lymphoid organs is associated with better anti-tumor immunity in lung cancer (85). Though the remodeling of lymphoid organs contributes to the anti-tumor immunity, some studies found that lymphotoxin derived from B cells supports tumor growth. Androgen promotes prostate cancer (CaP) growth by binding to the androgen receptor expressed on both normal and cancerous prostate cancer cells. Androgen ablation by castration induces cell death of cancer cells and lymphocyte infiltration in TME, and it is effective for androgen-dependent CaP patients, while many patients are castration-resistant (CR). B cells are abundant in TME of CaP, and the B cell-derived lymphotoxin in TME activates IKKα, which is involved in nuclear factor κB (NF-κB) signaling and promotes metastasis, and STAT3, leading to CR-CaP and prostate tumor growth (86, 87).

In addition, a recent study found that γ-Aminobutyric acid (GABA) derived from B cells promotes tumor growth by facilitating IL10^+^ macrophages in TME (88). In the study of MC38 colon cancer cell line inoculation *in vivo*, which is reported that B cells suppress anti-tumor T cell responses in this cell line (89, 90), and B cells secreted GABA promotes tumor growth by facilitating IL-10 production from macrophages. Though GABA production is not restricted to B cells, GABA production from B cells is much more than other immune cells in draining lymph nodes. In addition, B cell-specific GABA depletion restored anti-tumor immunity (88). Therefore, the metabolism network of tumor-infiltrating immune cells could be a valuable target for therapy.

## Discussion

**Figure 1:**
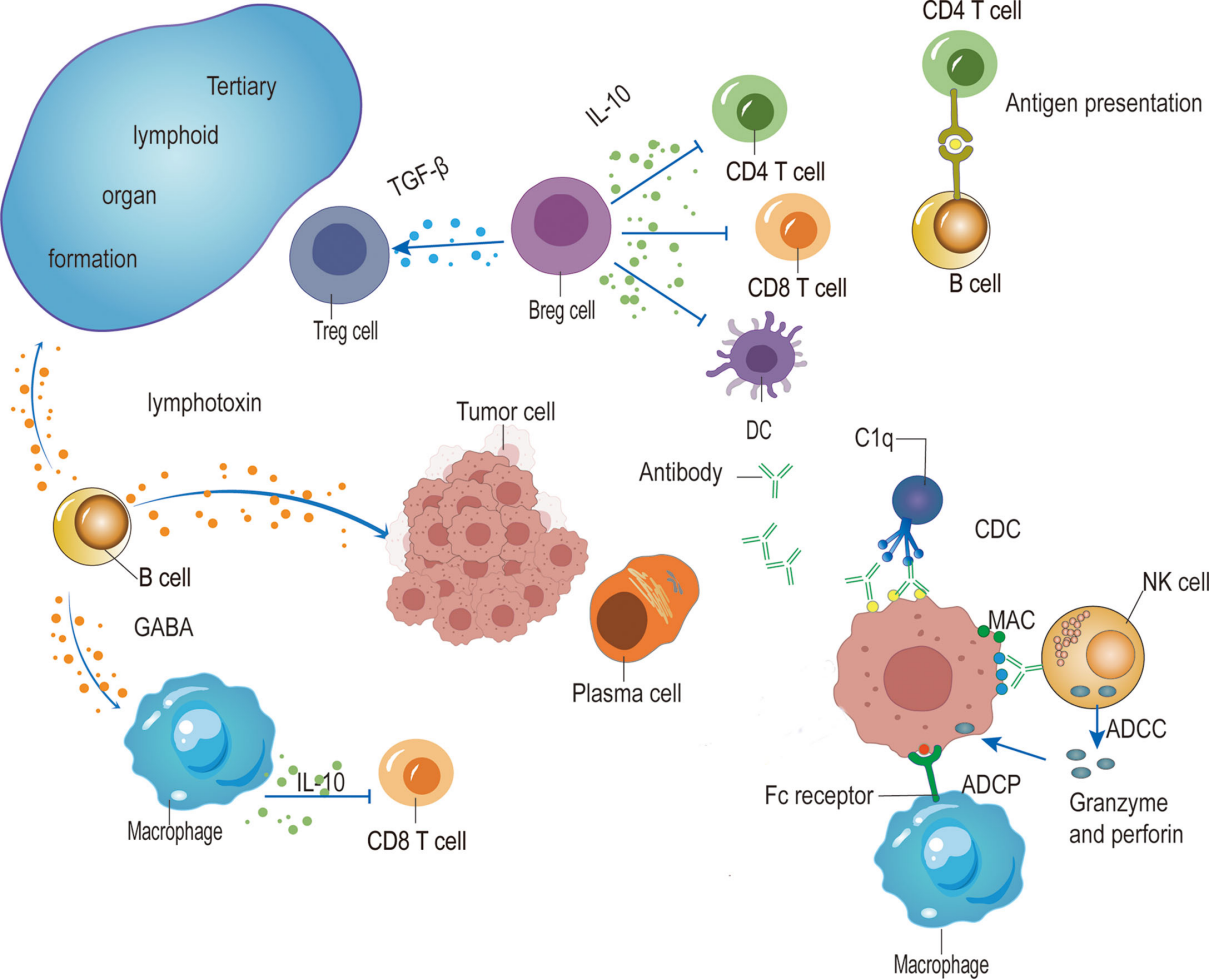
The role of B cells in tumor immunity. The antibodies produced by plasma cells induce ADCC mediated by NK cells, ADCP by macrophages, and CDC mediated by C1q, which target and kill tumor cells. IgA-expressing Breg cells dampen anti-tumor immunity by secreting anti-inflammatory cytokines such as IL-10 and TGF-β to suppress CD4^+^ T cells, CD8^+^ T cells and dendritic cells (DCs), and facilitate Treg cells. B cells also promote anti-tumor immunity by presenting antigen to CD4^+^ T cells and further interacting with activated T cells to induce TFH cells, thus promoting the function of CD8^+^ T cells. In addition, the production of lymphotoxin from B cells enhances anti-tumor immunity by facilitating tertiary lymphoid organ formation while promoting tumor growth by the induction of angiogenesis. Moreover, B cells produce GABA to impair anti-tumor immunity by facilitating IL-10-producing macrophages.

The function of B cells in cancer development is controversial. Different B cell phenotypes play a different role in various cancer (**Figure 1**). When the tumor cells express neoantigens containing BCR epitope, B cells can present these neoantigens and interact with neoantigen-activated TFH cells to facilitate the cytotoxicity of CD8^+^ T cells. Activated B cells further differentiate into ASCs. The IgG antibodies secreted by ASCs induce ADCC, ADCP, and CDC to promote anti-tumor immunity. Immunosuppressive IgA production in TME supports tumor growth. In addition, CIC accumulation is associated with poor outcomes. IL-10^+^ IgA-producing B cells could be categorized as a part of Breg cells, which suppress the anti-tumor immunity, other Breg cells such as TGF-β-producing B cells or IL-21-producing B cells also limit anti-tumor immunity. Moreover, B cells-derived lymphotoxin supports lymphoid organ development but promotes tumor growth and relapse by inducing angiogenesis. And GABA produced by B cells in TME impairs tumor growth by supporting IL-10^+^ macrophages.

Though there are many controversial functions of B cells in tumor immunity, the role of B cells in different tumor types is different. Therefore, it is still possible to look for an adequate B cell-based therapy in some specific tumors. For example, IgA^+^ Breg cells express PDL1, secrete IL-10 in TME and suppress local immune responses in several cancer types, such as human prostate and liver cancer (91, 92). PD-L1/PD-1 blockade can restore the anti-tumor immunity by reactivating CD8^+^ T cells since Breg cells suppress CD8^+^ T cells by producing anti-inflammatory cytokine IL-10. Simply depleting B cells couldn’t well demonstrate the function of B cells in a specific tumor cell type, thus, further studies may be needed to elucidate which phenotype of B cells or which mechanism is predominant. Yet, if the depletion of B cells largely impairs tumor growth, it can still be considered a potential treatment. Breg cells play a critical role in suppressing tumor immunity in some cases. Therefore, for these tumor cells, it is valuable to deplete Breg cells. However, since there is no good marker for Breg cells, it is challenging to deplete Breg cells specifically. In the case that B cell deficiency promotes tumor growth, antibody production, and antigen presentation might be essential. Therefore, B cell activation seems feasible in those BCR epitope-containing neoantigen expressing tumor cells. Though STAT3 activation and CD5^+^ B cell proportion are correlated with poor outcomes in B16 skin tumor cell lines (93, 94), adoptive transfer of activated B cells in tumor cell inoculated mice leads to slower tumor growth (95).

In summary, increasing studies found that B cell-targeted therapy could be a prospective candidate in immunotherapy. However, based on the mouse experiment, B cell-targeted therapy may not be as efficient as T cell-based therapy. Therefore, the combination of B cell and T cell-targeted therapy could be promising in cancer therapy.

## Author contributions

RT and WL drafted the manuscript. RT generated the figure. MN and WL revised the manuscript. WL designed the outline of the manuscript and revised the manuscript. All authors contributed to the article and approved the submitted version.

## Funding

This work is supported by Education Reform Research of Central South University (2022jy147).

## Conflict of interest

The authors declare that the research was conducted in the absence of any commercial or financial relationships that could be construed as a potential conflict of interest.

## Publisher’s note

All claims expressed in this article are solely those of the authors and do not necessarily represent those of their affiliated organizations, or those of the publisher, the editors and the reviewers. Any product that may be evaluated in this article, or claim that may be made by its manufacturer, is not guaranteed or endorsed by the publisher.
